# Book Reviews

**Published:** 1816-12

**Authors:** 


### CRITICAL ANALYSIS
### OF RECENT PUBLICATIONS
IN THE
### DIFFERENT BRANCHES OF PHYSIC, SURGERY, AND MEDICAL PHILOSOPHY.

---

*Medico-Chirurgical Transactions, published by the Medical and Chirurgical Society of London.* Vol. VII. Part I.—Longman and Co. 1816.

IT is not for a journalist to complain of the increasing expence of books. This, as well their multiplication, renders our labours the more necessary; still, we cannot help regretting, that either the supposed necessity of publishing half-yearly, or some other cause, should have induced the paper committee to devote nearly one-third of their expensive half volume to the title list of officers and members, a long paper on the waters of the Spa, and another not so long, but scarcely more interesting, on Angina Pectoris. It is but justice, however, to add, that many valuable papers follow. The greater number contain successful and unsuccessful accounts of operations absolutely necessary for the preservation of life, but so bold, that 'till of late years they would scarcely have been attempted by the most experienced veteran, or the hardiest candidate for celebrity. We shall, according to our usual custom, in order to keep up the reader's interest on each subject, separate the medical from the chirurgical papers, commencing with the first in their order.

*Chemical Analysis of the Mineral Waters of Spa;* by EDWIN GODDEN JONES, M.D.

We have no doubt of the accuracy and value of this paper; but our readers will not expect us to analyse an analysis, even should they permit the use of such a pun.

*History of Two Cases of Angina Pectoris;* by SAMUEL BLACK, M.D. of Newry.

"At the time, (says Dr. B.) when my first observations on the disease which has been named Angina Pectoris*, were communicated

---

"* The first of my papers is contained in the Fourth Volume of the Memoirs of the Medical Society of London, and was read to that Society, in March 1794; the second (Vol. VI.) was read in October 1796.

cated to the public, the affection was by no means so well understood, nor the morbid changes of structure on which it appears to depend so thoroughly investigated and ascertained as they have since been."

These remarks of the author may serve as our apology for passing over two cases, which however interesting, do not throw any new light on the pathology or treatment of a complaint arising, as Mr. Hunter remarked, from a great variety of causes.

*History of a very Fatal Affection of the Pudendum of Female Children;* by KINDER WOOD, Esq. Member of the Royal College of Surgeons, and Surgeon at Oldham.

This is a very interesting and a very important paper. The disease appears to be one of those epidemics which occur in camps, fleets, and other situations in which the human race is crowded. It assumes, as we observed when noticing Mr. Makesey's paper on Ophthalmia, all the various forms of general disease, as low fever, scurvy, or cutaneous eruptions, or of local disease, as erysipelas, sometimes in a manner (which, but for the certainty of the fact, we should hardly conceive) confined to particular parts of the body, and showing itself by ulcerated legs, ophthalmia, erysipelatous cheeks, or vesicles about the hands or about the genitals. Astruc first noticed the latter in a fleet, denominating the disease *christallines*, and from the part on which they appeared considering them venereal. We suspect they are more common in civil life than is generally suspected.*

All these diseases are attended more or less with fever, and, if neglected, or the patient is deprived of wholesome diet and air, frequently end in sordid ulcers or gangrene. The description given by Mr. Wood is very correct in all these points. We shall transcribe only the following as connected with an important question in medical jurisprudence. The reader will, perhaps, urge, that, in this instance, the patient was well in her general health until the local symptoms were manifest. But we must always make large allowance for the rank in life of our patient. The early or slighter complaints in the children of poor manufacturers in Manchester are very likely to be overlooked by the parents and even by the children as soon as they are old enough to be constantly employed in earning their daily bread. No wonder, therefore, if the child's indisposition attracted no notice

* See an ingenious paper by Mr. Royston in our Vol. xxiii. p. 241.

till the local symptoms were so violent as to make her complain.

" 'Jane Hampson*, aged four, was admitted an out-patient of the (Manchester) Infirmary, Feb. 11, 1791. The female organs were highly inflamed, sore and painful; and it was stated by the mother, that the child was as well as usual till the preceding day, when she complained of pain in making water. This induced the mother to examine the parts affected, when she was surprised to find the appearances above described. The child had slept two or three nights in the same bed with a boy fourteen years old; and had complained that morning of having been hurt by him in the night.

" 'Leeches, and other external applications, together with appropriate internal remedies, were prescribed; but the debility increased, and on the 20th of February the child died. The coroner's inquest was taken, previously to which the body was inspected, and the abdominal and thoracic viscera were found to have been free from disease. The circumstances above related having been proved to the satisfaction of the jury, and being corroborated by the opinion I gave, that the child's death was occasioned by external violence, a verdict of murder was returned against the boy with whom she had slept. A warrant was therefore issued against the boy, but he had absconded, a circumstance which was considered as a confirmation of his guilt, when added to the circumstantial evidence alleged against him.

" 'Not many weeks however had elapsed, before similar cases occurred, in which there was no reason to suspect that external violence had been offered; and some in which it was absolutely certain, that no such injury could have taken place. A few of the patients died, though from the novelty and fatal tendency of the disease, more than common attention was paid to them. I was then convinced I had been mistaken in attributing Jane Hampson's death to external violence; and I informed the coroner of the reasons which produced this change of opinion. The testimony I gave was designedly made public, and the friends of the boy, hearing of it, prevailed upon him to surrender himself.

" 'When he was called to the bar at Lancaster, the judge informed the jury that the evidence adduced was not sufficient to convict him; that it would give rise to much indelicate discussion, if they proceeded on the trial; and that he hoped, therefore, they would acquit him without calling any witnesses. With this request the jury immediately complied.' "

The following is the result of the author's own practice.

" Case I.—On January 22, 1815, I was desired to see Miss R. aged six years: she had complained three or four days of head-

" * See Medical Ethics, by Dr. Percival. Note by Mr. Ward, of Manchester, page 231.

ache; had been chilly, and occasionally hot; she had been sickly, and taken little food; was dull, heavy, and languid. This morning she had complained of pain in making water: upon examination the pudendum was found inflamed; upon which I was called in.

"The inner surface of the left labium was ulcerated, as well as the clitoris; the right labium was inflamed, and the whole parts tumefied, of a dark purple hue, not unlike some kinds of erysipelas; the mons veneris was enlarged and inflamed; the perineum was inflamed and covered with aphthæ, which also encircled the anus, the discharge was thin, copious, and offensive, and had inflamed the top of the thigh, where it had been suffered to remain. The face had a peculiar paleness; the bowels were slow; the pulse quick and weak; the appetite diminished; the tongue of a dull clay colour. She was thirsty, complained of chilliness, and was indisposed for motion. The liquor plumbi acetatis dilutus was ordered as a lotion, to be applied lukewarm; and poultices made up with the same fluid were directed. A decoction of bark was also given with confectio cardiaca.

"By the use of these means, the enlargement of the parts gradually subsided, the foul bottom of the sores became red, after which the ointment of white lead was used, and the parts healed by the 14th of February, a space of seventeen days from the first attendance.

"In this case the affection again returned, but was early cured by resorting to the same remedies. The patient frequently retained the urine twenty-four hours, the pain was so violent, and obstinately resisted every inclination to empty the bowels, so that the opening remedies were obliged to be exhibited with a regular attention.

"Case II.—On the 25th of April, 1815, I saw Miss S. aged five years and a half. She had been unwell a few days previous to the 21st, when complaining of pain in voiding the urine, the parts were examined and found slightly red; they were washed with milk and water, and dusted with the lapis calaminaris. On the 22d, the inflammation had increased, and the parts were slightly excoriated. On the 23d, a thick yellow discharge was observed, the patient was getting more unwell, the bowels were slow. On the 24th, the open surfaces were enlarging, and small watery vesicles appeared upon the labia and perineum; upon the left thigh also was a large cluster; the bowels were twice opened this day by some family purgative.

"On the 25th, I saw the patient, and found both labia enlarged, and of a purple redness, with numerous small watery vesicles, upon the external surface, and also within the fissura magna. They were similar to cowpock vesicles of the third and fourth day; were found also upon the perineum, and the top of the left thigh. In some places the tops of the vesications were loosened, and showed beneath a deep foul ulcer, particularly in the cluster upon the thigh, and on the anterior part of the labia. The parts within within the fissura magna were every where red and inflamed, and several small ulcers were found. The skin around the anus was painful and red; and the secretion was then copious and offensive. There was a dull headache, a quick and irritable pulse, a moist tongue, but bearing a clay-coloured deposit; the motions on the 24th, were dark-coloured and offensive; the patient was considerably weakened, and the face of a peculiar paleness. I advised saturnine lotions slightly warmed, and saturnine poultices without oil, to the parts, and gave small doses of pulv. rhei in a saline mixture every three hours.

"April 26.—Fresh vesications still appearing; and, when the tops of the earlier vesicles had come away, the parts beneath were deeply ulcerated. Several aphthæ were observed within the labia, upon the perineum, and around the anus. The skin was hot and dry; the bowels open, and motions dark and offensive; with excessive pain upon voiding the urine.

"27.—The top is thrown off from the cluster of vesicles upon the thigh, as well as from the vesications upon the pudendum and perineum; the open surfaces are deep and foul, secreting largely a thin and offensive matter; the anus surrounded with aphthæ; the pulse 120; skin hot and dry; bowels open, and urine excessively hot and painful. Ordered a decoction of bark, with conf. cardiaca; recommended a little red wine to the patient, and to continue the applications to the parts.

"28.—The bowels slow; the urine has been retained thirty hours; the abdomen tender and hard; with much difficulty she was prevailed upon to void the urine, which was copious, high-coloured, and of a strong smell: the aphthæ had almost disappeared, and the diseased parts shewed a large ulcer of various depths, extending over the pudendum and perineum, down to the anus; the parts within the labia were in the same state, and a deep ulcer, but not extensive, lay upon the left thigh, on its upper and inner part; the secretion is thin, copious, and offensive. The sores were ordered to be washed with the lotion as usual, and dressed with the white lead ointment. Continue the bark mixture, and increase the wine.

"29th and 30th.—The ulcerations were stationary; the same means were continued; but, as the bowels were slow, they were moved with an infusion of senna.

"May 1.—Sores improving; the bottom becoming less foul, and discharge less offensive; pulse 90 and weak; appetite poor; sits up a little; great inclination to retain the urine; bowels open. Continue the applications and remedies.

"2d.—The sores improving, as well as the strength. This state of improvement continued regularly, till the sores were healed on the 14th. After the healing, the pudendum continued discoloured and tender, and a considerable yellow mucous discharge continued with varying quantity for the space of six or eight weeks; this was relieved by a continued use of the tinct. lyttæ, bathing the parts frequently in the day with a solution of the sulphas zinci; the showers shower-bath was also used with the intention of checking the secretion, as well as getting up the strength. I saw this patient on the 27th of June, when the discharge had ceased.

"Upon looking over my notes, I find that, in nine years, I have seen twelve cases; of these, I have only seen the two above related so early as to be materially serviceable; the others, being among the children of labourers, had little chance, either from the attention or punctuality of the parents, of getting over so very formidable a disease. One, a little girl of two years old, recovered, and was attacked again in the course of a fortnight, which second attack proved fatal. In a girl, five years of age, where the earlier appearances of the disease had been entirely overlooked, the mother upon finding an extensive ulcer, brought the child to me, under the idea of its having received injury from fire, which had escaped attention. The case proved fatal."

Thus, of twelve cases, ten were among the labouring poor. By what means these epidemics reach the rich, cannot always be ascertained, but probably the greater matter of surprise should be that it does not oftener occur. Among children, it is still more likely, as it is impossible to prevent nursery maids from introducing their little charges to their own relations, and even in infantile day-schools the gradations must sometimes intermix.

*Cases and Observations illustrating the Influence of the Nervous System in regulating Animal Heat*; by HENRY EARLE, Esq. Assistant-Surgeon to St. Bartholomew's Hospital, and Surgeon to the Foundling Hospital.

"Previous to the interesting experiments published by Mr. Brodie, in the Philosophical Transactions for 1811, it was a generally received opinion, that animal temperature depended on the chemical changes which the blood undergoes in the round of circulation. On this supposition a beautiful and apparently satisfactory theory had been constructed. This much esteemed fabric of human reasoning has, however, received a severe shock from the experiments above alluded to, which tend to establish the following facts: that, when the brain has been destroyed, animal heat ceases to be generated, notwithstanding the functions of respiration are artificially continued, and apparently all the chemical changes are produced in the lungs; and further, that an animal thus subjected to artificial respiration, cools more rapidly than one that is simply killed by decapitation, probably in consequence of the circulating blood being exposed to the cold stream of air which is introduced into the lungs. From these facts it appears that nervous influence is essential to the production of animal heat."

Mr. Hunter has been often accused of reading little. If such was the case, ample revenge has been taken of him, as he seems not to be read at all. We have only mentioned the the name of Hunter, because that name is in every surgeon's mouth—him they commemorate by an annual festival. In honor of him, a magnificent mausoleum has been erected, and lectures instituted, as if in imitation of those athletic games by which the virtues and prowess of ancient heroes were celebrated. Did then "this much esteemed fabric of human reasoning receive no severe shock"* from his observations? (we avoid the word experiments for reasons which will presently appear.) Let us transcribe the following passage, published a second or third time only a year before his death.

"This power of generating heat, seems to be a property in an animal while alive. In the most perfect animals it is to preserve a standard heat; and as they are most commonly in an atmosphere colder than themselves, they have most commonly occasion to exert it; and it is therefore a power only of opposition and resistance; for it is not found to exert itself spontaneously and unprovoked; but must always be excited by the energy of some external frigorific agent, or disease; yet it is natural to such animals that this power should be called forth; as will be observed by-and-by. It does not depend *on the motion of the blood, as some have supposed*, because it likewise belongs to animals who have no circulation: and the nose of a dog, which is always nearly of the same heat in all temperatures of the air, is well supplied with blood; although we must allow, where this power is greatest, the circulation is the quickest: neither can it be said to depend upon the nervous system; for it is found in animals that have *no brain or nerves*. However, it must be allowed, that all that class who possess this power in the highest degree, have the largest brain, although this power is not in the least in proportion to the quantity of brain in that class. It is most probable that it arises from some other principle; a principle so connected with life, that it can, and does, act independently of circulation, sensation, and volition; and is that power which preserves and regulates the internal machine. This power of generating heat, is in *the highest perfection when the body is in health;* and in many deviations from that state, we find that its action is extremely uncertain and irregular; sometimes rising higher than the standard, and at other times falling much below it. Instances of this we have in different diseases, and even in the same disease, within very short intervals of time. A very remarkable one fell under my own observation, in a gentleman who was seized with an apoplectic fit; and, while he lay insensible in bed, covered with blankets, I found that his whole body would, in an instant, become extremely cold in every part, continuing so for some time; and, as suddenly, would become extremely hot. While this was going on alternately, there was no sensible alteration in his pulse for several hours.

"Being

"Being satisfied of the foregoing fact, that animals had a power of generating heat, I pursued the subject still further; not so much with a view to account for animal heat, as to observe the different phenomena, with the variations or difference in the heat in different animals."*

By the above passage it appears that Mr. Hunter proved, without the uncertainty of experiments, that animal heat does not depend "on chemical changes which the blood undergoes in the round of circulation;"—that it does not depend "on the nervous system:" but on that power which preserves and regulates the machine, and which is only influenced, not generated, by external circumstances, under health and disease. Consequently, that this great philosopher made his experiments, not with a view to account for animal heat, (for this would not have been less futile than the attempt to inquire into the principle of life,) but with a view to *observe the different phenomena*, with the variations of difference in different animals.

It is true, we are referred to an experiment [an *experimentum crucis*], in which, after the brain was removed, respiration being kept up artificially, animal heat ceased to be generated. If, indeed, we could suppose, that the power which regulates the "internal machine remains unimpaired," when the brain is destroyed, we might then say—

"The times have been,
"That when the brains were out the man would die,
"And there an end, but now they rise again
"With twenty mortal murders on their crowns,
"And push us from our stools. This is more strange
"Than such a murder is."

It certainly would have been strange, if either the generation of heat, or the secretions, had been regularly maintained, or reduceable to any laws in such a condition of what is called a warm-blooded animal; or, to use Mr. Hunter's more accurate expression, in an animal capable, under common circumstances, of preserving a standard heat.

Respecting Mr. Earle's paper, we ought to remark, that it contains many judicious observations. The result of some of his experiments surprised us; but so many particulars are requisite in every experiment connected with the operations of a living animal, that we are not disposed to doubt his accuracy, though the result does not always accord with our own observations. This is only in a single instance; most of

* Animal Economy, page 103, edit. 1792.

the the rest confirm a law established long since by Mr. Hunter, that, though, to the feeling, inflammation seems considerably to encrease the temperature of a part, yet, by experiment with the thermometer, the change never exceeds a very few degrees. It is not difficult to account for this. We are accustomed to make our remarks on inanimate matter, which, at first, raises or reduces our own temperature to its own; but, in a little while, the temperature of the two substances in contact is similar. Now, in touching a living body under fever, each part in contact maintains, as far as possible, its own standard. Hence, the encreased heat of the person under fever, is restored as fast as it is communicated to a person in health, and thus gives him the sensation of a much higher temperature.

We perceive Mr. Earle uses the term *arterious* for the adjective of artery. As far as we have had time to consider the subject, we suspect he is right; but we should be very thankful to learn his motive, as nothing evinces our improved philosophy more than a greater correctness of language.

*(The other Articles in our next.)*

*A Treatise on Uterine Hæmorrhage.* By DUNCAN STEWART, Physician-Accoucheur to the Westminster General Dispensary, and Lecturer on Midwifery, in London. 8vo. pp. 151.—T. and G. Underwood.

THOSE only on whom such a task devolves can be aware of the difficulties which beset our critical department. In vain do we resolve to confine ourselves to simple analysis, in vain attempt, by mere extract, to show the spirit, tendency, and merits of a work. Even if our readers expected no more of us, we find it impossible to restrain our feelings, if not of admiration or disgust, at least of surprise; and, of all the works which have ever appeared before us, we are ready to acknowledge the latter sentiment has been the most strongly excited by this Treatise.

In the advertisement prefixed we are told—

"The object of the following Treatise is to point out a mode of treatment, which has been found very beneficial in alarming cases of uterine hæmorrhage.

"In the Introductory Observations, an attempt is made to explain the functions of the uterus, by tracing the resemblance which that organ has in its action to the other involuntary muscles; and the remarks on this subject will not, perhaps, be thought misplaced, when it is considered that many of the best-established rules of practice in midwifery, have been the result of what is known of the structure and functions of the uterus.

"In the medical treatment of uterine hæmorrhage, I am not aware that opium is generally given, although it has been recommended by some foreign authors as highly beneficial. Doctor James Hamilton, Professor of Midwifery in the University of Edinburgh, to whom I am happy in having this opportunity of expressing my obligations for his instructions, and my respect for him as a practitioner, has long recommended it as the best remedy for relieving the irritation and state of debility which are induced by this disease; and Mr. Burns, of Glasgow, has also mentioned it as useful in this complaint. But, in many of the best publications on midwifery, the use of opium in uterine hæmorrhage is altogether condemned.

"The cases detailed in the following Treatise will, I hope, remove the prejudices which theory may have advanced against the use of opium in uterine hæmorrhage, and establish the good effects to be derived from giving large doses of that medicine.

"*Golden-square; Aug.* 1, 1816."

It must appear strange that men who have devoted a long life to midwifery, in this immense metropolis, should have been so deficient as to leave the most important part of the art to be elucidated by a gentleman recently arrived among us: for, though that gentleman is already a teacher, yet, as his book is dated 1st August, as his name is not in the College List published the last day of September, we must conceive either that he has arrived too recently to complete his examinations, or that he is under the age required by the College for a license. We take no notice of the want of M.D. to his name, as all our readers know such a title may be readily acquired, and almost at any age.

To return to the advertisement. Are none but foreign authors, and Dr. Hamilton of Edinburgh, and Mr. Burns of Glasgow, to be considered. Have no other writers given their opinion on opiates in these cases. If the author is "not aware that opium is generally given," would it not have been respectful to enquire of the London faculty what the usual practice is, or to have remarked that, after forty years of extensive practice, the venerable Denman had not indeed condemned or shewn any *prejudice* against it, but, from the result of long experience, with becoming modesty, given it as his opinion, that, unless with a view of moderating some uncommon degree of pain, or quieting some tumult, he seldom used opiates in abortions, considering the recurrence of pain as, in some degree, necessary for the proper contraction of the uterus.

The introduction contains the usual doubts and arguments relative to the muscular properties of the uterus. Among the rest, that there are other muscular parts in which we

We can distinguish no fibres. In an instant, fibres of a florid colour are supposed to be considered as necessary to constitute a muscle; and it is urged—"Nor has the existence of muscular fibres in the urinary bladder been disputed, although they are equally destitute of a florid colour." Are then colourless fibres equally evident in the uterus? We are told, too, that "when the uterus has once been ruptured, it is more liable to a similar *action*, in all future endeavours to expel the fœtus." How many cases, we would ask Mr. Stewart, has he seen of ruptured uterus? how many of second ruptures? or how does he calculate this increased liability to rupture?

The next paragraph we think it right to transcribe, that the venerable name of John Hunter may not be hacknied whenever an author wishes to escape a difficulty, by the introduction of a term, even though unsanctioned by such authority.

"The uterus, like the other involuntary muscles, is capable, not only of contraction, but of retaining itself in a contracted state, till that particular change in the economy of the parts takes place, which requires its relaxation. If it remain contracted longer than any other muscle, it is merely because the stimulus of relaxation, to use the language of Mr. John Hunter, is not applied; for, when this stimulus is applied, the uterus, like the other muscles which surround cavities, immediately obeys, relaxing, and gradually adapting itself to the changes which are going on."

We confess ourselves at a loss completely to understand the above. Does it mean that the uterus, when unimpregnated, is in a state of permanent contraction; or that the impregnated uterus is its uncontracted state? In other words, that the impregnated uterus is only that state of a muscle in which it is relaxed by ceasing to contract, and elongated by some other power. Such is the language of Mr. Hunter, when he speaks of the contraction of the rectum as the stimulus for the relaxation of the sphincter ani; adding that when that contraction in the rectum ceases, the sphincter again contracts. But what analogy has this to the altered size and figure of the impregnated uterus. When the sphincter ani is relaxed, its fibres are elongated, and its substance rendered thinner. The uterus, as it enlarges its cavity, instead of losing its thickness, usually increases it.

The first section contains, "Observations on the practice generally employed in Uterine Hæmorrhage." In this, after a few remarks on the manner in which other vessels usually act under hæmorrhage, the author proceeds—

"*First*, to point out wherein the plan of treatment, which has generally been recommended in uterine hæmorrhage, is defective;

 "And

"And next, to propose a mode of treatment which has been found very successful, and has resulted from considering the structure and functions of the uterus."

Here is evidently a want of caution in the use of words, or in the manner of arranging them, because it is impossible Mr. Stewart could mean to insinuate that his predecessors, in their mode of treatment, had not considered the structure and functions of the uterus; yet the casual reader might be left with that impression. If, however, we admit what the author remarks, we shall be obliged to allow that he has examined the uterus and its contents in a different manner from any of us.

"The vessels (says he) connecting the placenta to the uterus are very large, particularly the veins, which inosculate freely, and are without valves."

We are at a loss to conceive that the author can refer to vessels connecting the placenta with the uterus as very large, which have never yet been discovered, and into which the finest injections can never be forced; yet his language seems to imply nothing less.

In considering the practice *generally* employed, Mr. S. proceeds—

"The causes of these cases of uterine hæmorrhage, which take place before or in the beginning of labour, at the full period of pregnancy, have been divided into *unavoidable* and *avoidable.* In the manual treatment of those cases of the disease which arise from the first cause, medical men have nearly agreed; but the practice almost universally recommended, and generally adopted, when the disease arises from accidental detachment of the placenta, does not appear rational: and, although it has received the sanction of eminent men, whose opinions are entitled to the greatest respect, yet this ought not to satisfy those whose duty it is to think for themselves; and they do not deserve well of the profession, who rest satisfied with what others have done, without inquiring whether the practice which has been recommended may not admit of some improvement.

"When uterine hæmorrhage arises from the accidental separation of the placenta, the practice generally recommended is to puncture the membranes as soon as possible; and it is stated that this of itself, without any further interference, will almost always check the discharge, so as to remove all apprehensions of danger, till the child is expelled by the natural efforts."

"Before the practice, therefore, of puncturing the membranes (continues our author) in uterine hæmorrhage is adopted, the three following questions ought to be maturely considered:

"*First;* Whether, by puncturing the membranes before the os uteri is dilated, we retard or accelerate the delivery of the child?

"*Secondly*;

"*Secondly;* Whether, by puncturing the membranes before the os uteri is dilated, we can depend upon it as a certain immediate means for suppressing the hæmorrhage?

"*Thirdly;* Whether, by puncturing the membranes before the os uteri is dilated, we do not often destroy the chance of saving the child's, and sometimes even the mother's, life?"

The author then proceeds to show that, if the liquor amnii* escapes before the os uteri is dilated, a protracted labour follows; that, if the uterus contracts on the body of the child, though the hæmorrhage may cease, the delivery is likely to be protracted.

"But, (says he,) even granting that the uterus, by contracting, considerably diminishes the diameter of its vessels, as long as the child is alive, and still in the uterus, the fœtal mode of life will be continued, and in this respect the functions of this organ will remain unaltered: the quantity of blood circulating towards its vessels will, therefore, continue undiminished, their action will be necessarily increased, and the hæmorrhage will continue unabated. In this view of the subject, then, the evacuation of the liquor amni can be of little service in restraining uterine hæmorrhage."

"Many bad effects are said to arise from introducing the hand into the uterus; and some writers have even asserted, that to this cause may be traced many of the cases of cancer and phagedena of the uterus, which occur in advanced life. It is only, however, when the liquor amni has been evacuated, and the uterus has contracted firmly round the child's body, that introducing the hand can injure it. For, if the os uteri be cautiously dilated whilst the membranes are entire, the introduction of the hand can produce no bad effect, as no pressure will be made by it on any part of the uterus; and, when the child's feet are grasped and brought into the vagina, it will be turned with the greatest facility." &c.

We have made these long extracts to give the author fair treatment: and, if we had only to contend with a logician, we might be puzzled where to begin our answer, as it would be impossible for us to understand each other. The reader might, indeed, be amused, if words were all that are to be considered. But an infinitely more important duty awaits us. Without, therefore, doing more than quote the author's words, we shall take the liberty to ask what writers, or what practitioners, he refers to, who assert that phagedæna and cancer uteri, in advanced age, have been caused by introducing the hand during a parturition many years before?

---

* We know not by what authority Mr. S. always writes liquor *amni*. We could enlarge our inquiries of this kind, if we had no greater objections to his work.

who who ever suspected these difficulties when the feet are brought into the vagina? or, lastly, who, under dangerous hæmorrhage, would be satisfied with mere rupturing the membrane without instantly preparing to deliver by art?

The author proceeds to "*some remarks on the practice generally recommended in uterine hæmorrhage from detention of the placenta.*"

"Uterine hæmorrhage (says he) occurs after the delivery of the child, either from the placenta being retained by want of contraction, or by spasmodic and irregular contraction of the uterus, or in consequence of a change in the structure of the placenta causing a morbid adhesion of it to the uterus."

In answer to this, we believe few people will doubt—that neither the want of contraction of the uterus, nor the spasmodic or irregular contraction of the uterus, are the causes of dangerous retention of part of the placenta, and that a change in the structure of the placenta is not necessary for its adhesion to the uterus: lastly, that every adhesion of these parts at such a time is diseased, so that the epithet morbid is superfluous, and likely to lead the reader astray.

"When hæmorrhage takes place from atony of the uterus, if the placenta has descended into the vagina, it has been recommended to leave it in this situation for some hours, as it is supposed to favour the permanent contraction of the uterus. When the inactive and debilitated state of the uterus, and the faint state of the patient, are considered, this practice will appear very dangerous. For, although the placenta prevents the blood from appearing externally, yet internal hæmorrhage may be going on, and the patient fast sinking before the danger is discovered. Many fatal cases of this kind are upon record; and the uterus has been found to have yielded, until it became, by being distended with blood, of a larger size than before the expulsion of the fœtus. No possible advantage can be obtained by leaving the placenta in the vagina; for, if the uterus has completely contracted, the hæmorrhage will be stopped; and if it has not, it is evident that the presence of the placenta in the vagina is not likely to promote its contraction, but to have an opposite effect."

We should be glad to know who recommends this practice, when hæmorrhage takes place from the *atony* of the uterus? or where those cases are recorded, which the author speaks of as so numerous, in which, by a subsequent distention from blood, the uterus has become larger than before the child was expelled? We have, indeed, heard of the ovum filling with blood after the rupture of the membranes, and thus deceiving the practitioner, who might expect that the hæmorrhage had ceased; but this is not very common, as the same introduction of the hand by which the membranes branes are ruptured, for the most part, serves for attaching the feet of the child. But such an atony of the uterus as is here described can hardly exist, excepting under circumstances in which the powers of life are so exhausted that no hope can remain of preserving the patient.

"Fainting, (we are told,) in these cases, is a most alarming symptom, especially if the uterus is in a state of atony: for the vessels which pour out the blood are so large, that the hæmorrhage generally continues in some degree, notwithstanding the patient's faint state: and, when she recovers, the sudden increase of the discharge will cause an immediate return of the syncope. But, if the hand be cautiously introduced, it gives little pain, and generally has the good effect of exciting the uterus to contraction, and likewise of communicating a degree of stimulus to the general system, which rouses the patient, so that she is able to swallow such substances as will contribute to her restoration.

"When the placenta adheres so intimately that the powers of the uterus are incapable of separating it, it becomes necessary to introduce the hand to assist in effecting the separation. The practice generally recommended in these cases, is to insinuate the fingers between the placenta and the uterus, and to peel it off. But, by following this plan, whilst the separation will be attended with great pain, and considerable risk of lacerating the internal surface of the uterus, the hæmorrhage will be increased, more vessels being lacerated; and the object of introducing the hand will be but imperfectly accomplished. When the placenta is converted into a cartilaginous or bony structure, it often adheres so intimately, that it will be impossible to tear it away without lacerating the uterus; and, when it is morbidly soft, by separating it in the method above mentioned, part will be left adhering to the uterus, which will be attended either with immediate or future inconvenience.

"Uterine hæmorrhage sometimes occurs after the placenta is delivered, and then it generally arises from want of contraction in the uterine fibre. In these cases, the long-continued application of cold has been generally advised; and this practice has been carried to the extent of immersing the patient for hours in ice-cold water. Cold, when applied suddenly, has the effect of exciting the uterine fibre to contract; but the long-continued application of it, if the effects on the general system are considered, cannot be expected to excite the uterus to contraction, and must otherwise prove injurious. Plugging the vagina has likewise been recommended in those cases of the disease; but this plan must be very dangerous,—for sometimes, when a coagulum of blood fills up the os uteri, an accumulation of blood takes place within the uterus, and the patient sinks from internal hæmorrhage, although no blood appears externally.

"From these remarks it will appear, that the plan generally recommended for treating those cases of uterine hæmorrhage which take take place after the delivery of the child, will admit of some modification."

Those who have perceived, in the writings of the modest Denman, the slow progress of obstetric improvement till it fell into the hands of practical anatomists; who have afterwards seen, in the same writer, the progressive advances it made during the practice of Sandys, Smellie, and W. Hunter; the manner in which they gladly improved by each others observations, and advanced by slow degrees!——we will say no more, but regret that short popular treatises, in which plausible attempts are made to render decision on delicate points easy, should become so frequent. Nothing can be more desirable than to establish certain aphorisms in all intricate cases. These are taught, with as much perspicuity as the cases will admit, in printed works; and we have happily hitherto possessed a succession of teachers in the metropolis, with every clinical assistance which this important branch of medicine can admit.—Without, therefore, noticing the above, we shall only offer a few specimens of the author's practice.

Respecting hæmorrhage in the early period of pregnancy, we are told—"When abortion is attended with profuse hæmorrhage, the discharge may easily be commanded by *stuffing* the vagina." In the more advanced stage of pregnancy, stuffing is objected to, and speedy delivery advised, to assist which, four grains of solid opium and 100 drops of laudanum are said to be very useful. This is a large dose for a London lady!

Among the directions, we shall copy the following:

"*As soon as* the child is expelled, the hand must be introduced into the uterus, and retained there, till that organ, by contracting, separates the placenta, and forces it into the vagina."

We say nothing of the evident impropriety of introducing the hand into the uterus, "as soon as the child is expelled," without waiting to discover whether such operation will be at all necessary. But, suppose, when the hand is introduced into the uterus, the operator perceives the flooding to be increasing, and finds that the presence of his hand neither restrains it, nor makes the uterus contract, which is no uncommon occurrence, ought he not to proceed to detach the whole of the placenta, the partial separation of which is the cause of the flooding?

We have said enough to caution our younger readers against what we conceive a juvenile performance, the publication of which will, we trust, induce the College to renew their former practice of examining licentiates in midwifery;

 and, and, perhaps, they may add a few additional questions to teachers of that important art. The following case we offer as illustrating Mr. Stewart's practice.

"I was convinced, (says Mr. S.) from the whole circumstances attending the case, that the only chance of saving her life consisted in the speedy delivery of the child; but, before proceeding to accomplish this purpose, eighty drops of laudanum were given, which, after waiting twenty minutes, produced no sensible effect. One hundred and twenty drops more were therefore given, which, in ten minutes, were followed by drowsiness, with a remission of the vomiting and tremors. At eight o'clock, the hand was introduced into the vagina, the os uteri cautiously dilated, the placenta detached at one side, the membranes ruptured, and the child's feet grasped, and brought into the vagina. The vomiting and restlessness again recurring, eighty drops of laudanum were given, which produced composure, and a permanent cessation of the vomiting. The fœtus, which appeared to be of the seventh month, was gradually extracted. The hand was introduced immediately afterwards, and the uterus contracted, separating the placenta, and forcing it into the vagina; from whence it was gradually removed."

It is easy to understand, by the above account, that the woman took 280 drops, rather more than half an ounce of laudanum, besides brandy and beef-tea, in the space of about an hour. That this may sometimes be safe, and even necessary, cannot be doubted; but should it be offered as a general rule? As to the manipulation, the account is somewhat more obscure.

Before we ventured to send the above to the press, we thought it right to consult a friend, who has been little short of forty years in practice, and who, from being one of the physicians of the most extensive Lying-in Charity in London, must have had a competent share of experience in the most difficult cases. From him, besides some of the above remarks, we learn that a preparation of equal parts of laudanum and dilute sulphuric acid, has been the remedy entrusted, with proper instructions, to the midwives, for their use in cases of hæmorrhage, perhaps from the first institution of the charity, but certainly for fifty years past. So that the use of opiates in uterine hæmorrhage is not new. Our friend informs us that he seldom exhibits opium in hæmorrhage, confining it almost entirely to such cases as are accompanied with irregular spasmodic contraction of the uterus, or with severe pain. His principal reason for abstaining from opium is one which Dr. Stuart uses in recommendation of it, viz. that it prevents fainting. Now, fainting our friend considers as always a desirable event, being nature's remedy for stopping the discharge, and one that never never fails, except its efficacy be counteracted by brandy, wine, or other cordials, of which opium may be reckoned one; and, to insure this effect, Dr. Stewart very frequently unites brandy with it. To the enquiry, what remedies he puts most confidence in for restraining uterine hæmorrhage? our friend mentioned two, in which, supposing the uterus to have been previously emptied of its contents, or that its evacuation was deemed improper, he nearly puts his sole confidence: these are, *cold water*, not as applied externally, but taken into the stomach; and extreme *quiet*, avoiding, by every possible means, the rousing the powers of life which Dr. Stuart is so constantly attempting by the exhibition of brandy and opium.

The College of Physicians have, in their list of licentiates, men of distinquished abilities, and, for a series of years, confined to the obstetric art; and they could very lately exhibit the names of Denman, Combe, and Clarke, as licentiates in midwifery. One of these still remains. Why then do they not establish a court of examiners, consisting of their president, aided by such men. We are aware such a court cannot, with propriety, be formed from their own members, strictly so called, who by charter or bye-law are interdicted from chirurgical practice. The examiners of the Surgeons' Company are scarcely more competent, as none of them are practising accoucheurs. Thus, whilst a court is established for the examination of apothecaries, surgeons, and physicians, the softer sex, under circumstances the most interesting in the animal creation, and in which two lives are at stake, are to be left at the mercy of those who chuse to undertake the care of them, and even to teach others.

In our last Number, we begged a truce to the obstetric controversy; but, should Mr. Stewart, or any of his friends, anonymously or otherwise, think proper to animadvert on this article, we shall not fail to do them justice.

---

*A Treatise on the Nature and Cure of Gout, comprehending a general View of a Morbid State of the Digestive Organs, and of Regimen; with some Observations on Rheumatism.* By CHARLES SCUDAMORE, M.D. Member of the London College of Physicians, and of the Medical and Chirurgical Society of London. pp. 402.—Longman, 1816.

DR. SCUDAMORE's researches are marked by a philosophical spirit. He has contrived to infuse a good deal of interest into his pathological discussions. We are to look, says he, both in principle and practice, not to an undefinable and and untangible something pervading the frame, but to the peculiar *actions* of the organs destined for secretion and excretion in connection with the state of the digestive functions: and it is to the disturbance or derangement of these last that every thing in the rationale of the disease in question is to be regarded as subordinate. His views, indeed, of gout, are confessedly those of Mr. Abernethy, in reference to general deviations from health; and we think he has carried his simplicity of doctrine to a *verba-magistri* extreme. We shall, however, present our readers with an analysis of the book, having, on former occasions, expressed our aversion to the modern affectation of composing original essays, partly extracted from the work which they pretend to review.

Instead of Dr. Cullen's division into *regular*, *atonic*, *retrocedent*, and *misplaced* gout, Dr. S. proposes to divide the disease into "*acute*," "*chronic*," and "*retrocedent*," considering the acute form of the disease, without regard to particular situation, as the one species, the chronic as the other species, and the retrocedent as the variety. The following are his definitions of these states:—

"*Gout.* A constitutional disease, producing an external local inflammation of a specific kind; the susceptibility to it often depending on hereditary bodily conformation and constitution, but more frequently wholly acquired, not occurring before the age of puberty, seldom under the age of five and twenty, and most frequently between the ages of twenty-five and thirty-five; affecting chiefly the male sex, and particularly persons of capacious chest and plethoric habit; in the first attack invading usually one foot only, and most frequently at the first joint of the great toe, but in its returns affecting both feet, the hands, knees, and elbows, not only in the articular structure, but also in the other textures belonging to the moving powers, different parts being affected together or in succession; often accompanied with sympathetic inflammatory fever, which is marked by nocturnal exacerbations, and morning remissions; much disposed to return at periodical intervals, and often ushered in by premonitory symptoms.

"*Acute Gout.* Inflammation and pain of the articular, tendinous, or bursal structure, usually attacking one part only at the same time, but, in succession of attack, affecting different parts together; with preternatural fullness of the adjacent veins, and, in certain situations, with œdematous swellings of the integuments, occurring in twenty-four or forty-eight hours from the invasion of the fit; vivid redness of the surface, which is sometimes shining; entire disability of the affected part, with peculiar sensations of burning, throbbing, cutting and pricking, and weight; the action readily changing situation spontaneously, or from slight causes; causes; terminating almost invariably without suppuration, and usually with critical indications of the event.

"*Chronic Gout.* Inflammation and pain more slight, irregular, and wandering, than in the acute; faint redness of surface; much permanent distention of parts, or continued œdema, and impaired moving power; without critical indications of its terminating; associated with a morbid state of the digestive organs, a languid or oppressed circulation, and much nervous irritation in the system.

"*Retrocedent Gout.* Metastasis, or transference of the gouty action in the paroxysm, from the external part to some internal organ."

When we allow that the above definitions are superior to those of Dr. Cullen, we must, at the same time, observe, that they are, in some measure, lengthened out into the shape of histories, and have, therefore, this advantage over the more concise and more properly speaking definitions of the celebrated Edinburgh professor. Our author objects to Dr. Cullen's description of *retrocedent* gout, inasmuch as it assumes the internal affection which takes place upon the retrocession of the outward inflammatory action, to be necessarily and invariably *atonic* or spasmodic; whereas it is something as decidedly inflammatory as that external affection of which it has proved vicarious. This exception may, upon the whole, be regarded as a salutary caution, since it is of the highest moment, in reference to practice, to distinguish between gouty spasms and inflammation; at the same time, it must be admitted that metastasis in gout is most common in those feeble and debilitated subjects who are more frequently the subjects of spasmodic than inflammatory action. In rheumatism, on the contrary, metastasis seems sometimes to take place with a readiness and rapidity proportioned to the state of high excitement under which the individual is found at the time of the attack.

Dr. S. presents his readers with the following statement of seventy-one examples, under his own observation, of *the parts affected in the first fit.* This we transcribe for the benefit of the young practitioner.

"In the great toe of one foot only, *forty-nine cases.*
"In the great toe of each foot, *four.*
"In the toe and instep, *two.*
"In the outer side of one foot, *two.*
"In one ankle, *two.*
"In each ankle, *one.*
"In the ankle and instep of one foot, *one.*
"In the toe, instep, and ankle, of one foot, *one.*
"In the instep of each foot, *one.*

"In

"In the heel of one foot, *one*.
"In each foot and hand, *one*.
"In one toe and thumb, *one*.
"In the right knee, *one*.
"In the left knee, *one*.
"In one hand at the back, *one*.
"In one wrist, *one*.
"In each hand at the back, *one*.

"From this statement, (says our author,) it appears that *pod*agra is too limited a term even to mark the first fit, as an appropriate designation."

With regard to the "bodily conformation" of the subjects of athritic affections, Dr. S. tells us, that they are "for the most part formed with a capacious and circular chest; that they have large full veins and loose solids." That the disease is more common to men than females every one is aware, "which must (says our author) be principally referred to the chief remote causes, excess in living, especially excess in wine, being applied in a greater degree to the former. But, in addition to this circumstance, the superior delicacy of the female structure and habit, puts some restraint on the acquirement of the inflammatory and plethoric state of vessels which appertains to gout. The actions of the uterus are not without effect in counteracting a general redundancy of blood."

Hippocrates, says Dr. S., observes, in one of his aphorisms, "that gout seldom occurs in women till after menstruation has ceased;" but we must remark a little want of correctness in the rendering of the aphorism in question, which merely asserts that females are not attacked with gout unless the menses be *deficient*, at least such is the sense which we have always been accustomed to attach to the verb *εκλείπω*, which is the word made use of in the aphorism referred to.

When, on the subject of predisposition, Dr. S. is naturally led to the question of the hereditary nature of gout; and, from his own observation, he concludes, that, although the malady is decidedly traceable in some instances from parent to progeny, yet that it is for the most part actually acquired without any hereditary tendency in the habit.

"Dr. Adams (he says) has drawn a distinction which appears to me not very well founded, between the disposition and predisposition to disease. He attaches the strongest signification to the former of these expressions, (for they are only expressions,) a signification, which, as the word is compounded, is assuredly rather due to the latter. The epithets of strong and slight, in connection with either expression, would, I conceive, make the distinction sufficiently clear and marked. The author (Dr. A.) observes, 'if it it were true in all, as it is in most, cases, that the habits of the sedentary and healthy are necessary to induce the gouty action, there could be no question that it is only hereditary in predisposition; but, in some, the susceptibility to gout is so strong as to require no other stimuli for inducing the action than such as seem absolutely necessary for the support of ordinary health.' In gout, therefore, we must admit the two degrees of susceptibility, *disposition* and *predisposition*, nor will it be often difficult to fix their exact limits. In his fundamental arrangement of the subject, the following view is offered. 'Diseases either appear at birth, in which case they are called *congenital* or *connate*, or they arise afterwards. The first can only with propriety be called *hereditary*, or *family susceptibilities*, to certain diseases.' This distinction of Dr. Adams's (continues Dr. S.) appears both judicious and necessary; but, probably, the reference in each case must be made to *structure*. For myself I confess that I cannot form any satisfactory notion of hereditary quality that is not founded on *structure*."

Whatever tends to the production of vascular fullness, is regarded by Dr. S. as a predisposing cause of gout.

"In this country, (he adds,) and particularly in the metropolis, gout is much increased in frequency, among the lower stations of life, since the very general and free use of porter. This he considers a very nutritious fluid, and, in conjunction with spirits, even with a moderate quantity of solid food, may be viewed as inducing the plethoric inflammatory state, and as a consequent introduction to gout. In Scotland, (our author continues,) gout is much more rare than in England. In Edinburgh, where the habits of the people approach the nearest to those of London, it is found most; but it is scarcely ever known among the inferior classes. In two thousand two hundred cases of disease admitted into the Royal Infirmary, as clinical patients, under the care of Dr. Gregory, there were only *two* examples of gout. I also learn that Dr. Hamilton, in the course of thirty years' attendance at the Infirmary, has not seen more than two cases of gout. In the London hospitals, gout is rather frequent. In the abstract of diseases admitted at St. Thomas's Hospital, during ten years, under the care of Sir Gilbert Blane, in which the total number stated is 3813, the proportion of cases of gout is 130."

We have already hinted that Dr. Scudamore is averse from those theories of gout which go upon the assumption of a specific matter diffused through the system as its immediate source. The appearances which the urine of gouty subjects exhibits have been thought, even by some modern authors, to be in favour of this assumption; but Dr. S. affirms that the sediments which commonly occur in the urine of persons labouring under gout "are neither necessarily nor regularly attendant on a paroxysm of gout, and that they are found under under other circumstances of disease, in connection with unhealthy chylopoietic functions. In proportion, therefore, as gout is connected with such disordered functions, *and not further*, are these urinary evidences connected with that disease."

An interesting passage follows on the component parts of urine. Dr. S. conceives that experimenters on the qualities and constituent parts of urine have been misled in not taking into account the different proportions of ingredients, according to the difference of its specific gravity. "An abundant deposit (he says) of gravelly crystals is not to be considered as a proof of an excess of uric acid, but rather as a *separation* of this principle from the urine, and a new combination with some other of its elements; and the deposition of the pink, or lateritious sediment (which is regarded by our author, contrary to the opinion of Proust, as formed principally of the same ingredient with the gravelly crystals), is invariably connected with a high specific gravity of the urine. It seems to us, however, that the author, on this head, is rather disposed to strain a point in favour of his chylopoietic hypothesis; for we believe, that in two given quantities of urine, of precisely the same specific gravity, from two individuals, the one only a gouty subject, and, we would further add, from individuals who should be, as far as could be ascertained, in the same condition in reference to their digestive organs, the *total* quantity of uric acid would be found much to vary, as well as the relative quantity and combination of other ingredients. Neither would there be necessarily in both either the gravelly deposition or the lateritious sediment. Again, the particularly acid nature of the perspiration, under a paroxysm of the gout, is denied by our author, with somewhat, we conceive, too much of hypothetical strictness; for, allowing, with Berzelius, that "the matter of transpiration is *always* acid, and reddens litmus paper very distinctly," yet we believe this acidity to be, in some cases of gouty paroxysm, much increased. We are not, by any means, disposed to regard these excretory peculiarities as proofs of gouty matter, according to the theory of some; but may we not suspect our author somewhat tainted with that disposition to theory of which he accuses others.

He admits, in another place, that "gouty inflammation is an external evidence of a morbid condition of the system," and even opposes Mr. Hunter's more probable supposition "that the gout is not *always* an act of the constitution, but that parts may be so susceptible, or rather disposed for this action, that they immediately run into it when deranged."

 Surely

Surely then, according to his own admission, there is something more necessary to the production of a paroxysm than mere disturbance in the chylopoietic functions; otherwise, these disturbances and derangements, upon which it is so much the fashion to lay the whole stress of affairs, ought invariably, and without exception, to bring on gout.

"Ligament (continues Dr. S.) is probably the texture which is the most frequent seat of the gout; but the bursæ mucosæ, the sheaths of tendons, and the muscular aponeurosis, together with the respective vessels and nerves of these parts, may be also enumerated as textures *primarily* affected. Secondarily, the cellular membrane and skin share in the effects of the inflammation. The textures just now mentioned, belonging to the functions of the joints, do not appear susceptible of the suppurative inflammation"—which is very unusual in gout. In one case which Dr. Scudamore relates of gouty abscess, the suppurative process took place wholly in the common integuments.

The diagnosis, or discrimination of gout from rheumatism, erysipelas, and phlegmonis, is not, for the most part, attended with much difficulty: we shall pass on, therefore, to the prognosis, which is said to be favourable, "when the *visceral organs* are sound in structure, and not materially disturbed in their functions; when the tongue becomes moist and clean; when there is a return of the natural appetite, the *fæces recovering a healthy character*, the urine ceasing to deposit sediment, and at the same time losing its high specific gravity; when the nervous system becomes tranquil; and when the local sensations readily yield in their severity to remedies, the inflammation soon abating, and not shewing a disposition to quick transference from one part to another, or, if it be fugitive, not fixing severely on new parts."

These two last symptoms we conceive of the greatest importance for the practitioner to take cognizance of, when forming his judgment of the severity and probable obstinacy of the malady; and it is with pleasing and unqualified approbation that we transcribe the following sentence of our author, bearing upon these points, which we are heretical enough to suppose even more important to fix the attention than the state of the digestive functions.

"Among the unfavourable signs in gout, (says Dr. S.) I consider the strongest to be a *quick transference of severe inflammation from one part to another*, joined with painful sympathy of the stomach or the head, and with exquisite sensibility of the whole nervous system."

Our author ushers in his dissertation on the treatment of gout by a condemnation of the principle either of leaving the disease entirely to nature, or encouraging its establishment, ment, upon the supposition of its being a salutary exercise of the *vis medicatrix naturæ*. He is not, however, a disciple of the Kinglake school, in regard to the fearless application of local remedies.

"I should assume it (says he) as a principle that we should attempt the prevention of a fit of gout, if warned of its approach, and interrupt its progress when formed, unless such a state of the constitution exist, that the gout has taken place of another more serious disease, or may be expected to prevent one which is threatening, and more to be dreaded than itself."

With regard to bleeding generally in gout, the local inflammatory action is, he thinks, best kept under by other means, and the circumstances of the frame are not, for the most part, such as to require or admit of this kind of depletion; but the objection to bleeding, he thinks, has been carried too far, and "a prejudice of very antient date has been established against taking away blood *generally* in the gout under any circumstances."

Emetics are disapproved of, unless an evacuation of the stomach in a full degree is obviously required: a case, however, is given, in which the preventive power of an emetic was abundantly conspicuous. The combination of saline purgatives with diuretics is highly extolled; and, by the following formula, used to such an extent as to procure from four to six evacuations in the course of the twenty-four hours, much good may be effected.

"℞. Magnesiæ ℈j.

Sulphatis Magnesiæ ʒj. ad ʒij.

Aquæ Menthæ viridis ʒx.

Aceti Colchici ʒj. ad ʒifs.

Syrupi Croci ʒj. M. fiat Haustus, quartis, sextis, vel octavis horis sumendus.

"This treatment should be actively pursued until the gouty inflammation subsides; and so long as the urine, *which is first passed in the morning*, retains a high specific gravity, or, as a rule of more easy application, so long as it deposits sediment. In proportion as improvement in these points is obtained, the repetition of the medicine should be lessened to twice or thrice in the twenty-four hours; but it should not be discontinued until all inflammation is removed, the fæces and urine acquire healthy characters, and the tongue becomes clean and moist."

Dr. Scudamore's observations on "mercurial preparations," we think very judicious; indeed the whole of his practical inferences and indications are marked by much accuracy of judgment, and discriminating good sense.

"Mercury, (he says,) when occasionally employed as a mild alterative, or joined, in a full dose, with purgative medicine, has a

 full full claim to our regard; but, when given in frequent doses, so as to excite mercurial fever, more or less of serious injury follows, as a certain consequence, without any corresponding advantages."

Against the pretensions of *specifics* for gout, especially against the claims of the *Eau Medicinale*, and other specifics, Dr. S. is very severe. He tells us, at the same time, that the alleged discoveries of the composition of this celebrated medicine are all fallacious. As this is a matter of much present interest, we shall transcribe the account which our author gives of his own experiments, in reference to the eau medicinale itself, and the several other compositions with which it has been erroneously identified.

"*Eau Medicinale.*—Colour and consistence similar to the extract of poppy; taste slightly bitter, and much resembling the extract of herbane; smell perfectly distinct from that of opium, and very similar to the common treacle lozenge; soon deliquesces after being dried.

"Mixture of *hellebore and laudanum.*—Colour and consistence similar to the eau medicinale; tastes strongly and smells slightly of opium; soon deliquesces after being dried.

"*Tincture of Colchicum.*—Colour lightly brown; taste slightly, but distinctly bitter, and entirely different from that of the eau medicinale; smell, that of gum resin of guaiacum; soon deliquesces after being dried.

"*Tincture of Hedge Hyssop.*—Colour almost black; taste very bitter, like *Taraxacum;* no distinct comparable smell; dried and exposed in a damp apartment, very slowly and scarcely deliquesces."

A detail of some serious and fatal consequences, which have followed the use of *eau medicinale*, concludes thus:

"The usual bad results which the eau medicinale produces are very slightly balanced by the few examples in which it has given continued satisfaction; and, unless its composition should become known, and then receive some useful modifications from combination with other medicines, and from union with more general principles of treatment, I hope it will be entirely discarded from the list of remedies in gout."

In the opinion of Dr. Scudamore, Peruvian bark and sudorifics may be, for the most part, superseded by purgative and sedative medicines: opium, judiciously employed, he strongly recommends; and, when idiosyncrasy prevents its full use, he has found henbane a useful substitute, "but it must be confessed, that, in severe pain, it is on opium alone that much dependance can be placed." The active virtues of the humulus lupulus, recommended by Freake, appear to our author "to be very questionable."

After these observations on the general treatment of gout, a consideration a consideration of its local remedies follows. They have never, he affirms, been established upon fixed and regular principles. Leeches are considered as of doubtful propriety, and occasionally injurious. Vesicatories, irritants, warmth, and pediluvium, are all condemned, as well as the cold application of Dr. Kinglake, upon which we meet with some severe animadversions. It is, however, allowedly necessary, while the attention is principally directed to constitutional requisitions, to allay the local irritation; and Dr. S. proposes to accomplish this object by the employment of the following lotion, of which he has the satisfaction to state, he has made trial in about forty cases with the best success.

"℞. Alcoholis ℥viij.

Misturæ Camphoræ ℥xvj. M."

This lotion is ordered to be made moderately warm, by the addition of some hot water, and kept constantly applied to the part inflamed.

"The evaporation (says Dr. S.) which the alcohol alone would occasion, is advantageously restrained by this dilution; and the addition of a sufficient quantity of hot water is for the purpose of producing a temperature just agreeably lukewarm, and furnishes a prompt and convenient method of employing the lotion, on the principles on which I recommended its adoption. If it be applied either hot or cold, the intention of the remedy is frustrated; and I have observed, that, from being made too warm, its operation has been injurious rather than beneficial. If the temperature is measured by the thermometer, I may state that it ought not to be less than 75, or more than 85. I consider, however, that the expression of *just agreeably lukewarm* is a secure and sufficient direction to the patient. The linen compress, constantly kept wetted with the lotion, should consist of several folds, and the slightest and coolest covering only should be superincumbent."

Several judicious instructions follow on the management of the convalescence: of these our limits will not permit us to give but a very slender abridgment. "It is not sufficient," observes Dr. S. "that our treatment has been active in the paroxysm: we have a great and two-fold duty remaining to be performed—the restoration of the healthy state of the digestive functions, and of due strength in the weakened limbs." The tincture of ammoniated iron, in such cases and circumstances as do not forbid the use of steel, from too much vascular fullness and action, "may be taken advantageously in warm water twice a-day, in doses of twenty drops, gradually increased to sixty; joining with its use, as occasion requires, a suitable dose of the pulvis aloes compositus, formed into a pill with a decoction of the same and a little soap." General alteratives; moderation in diet; change

 of of air; the use of a circular roller, either of flannel or cotton, to œdematous limbs; and sponging the parts with tepid salt and water, with the occasional employment of a stimulant liniment, complete the catalogue of remedies recommended to assist the convalescence; subsequently to which our author details a series of instructive cases, in the relation of which he intermixes some valuable pathological observations. Through these, however, we have not space or leisure left to accompany him; nor can we find room for an analysis of his prophylactic rules of regimen, the general tenor and tendency of which may be easily inferred from what has already been advanced. His principles, of course, lead him to question the specific anti-arthritical properties of magnesia and the alkalies, respecting the former of which, he asserts, that it appears to him to be no further deserving of dependance than as an useful auxiliary to more active and comprehensive means.

Dr. Scudamore's formulæ are, for the most part, neat and judicious; but not always so consistent with the principles of chemical union as we expected from a writer who has proved himself a considerable adept in chemical science. His language is easy, unaffected, and perspicuous; and the work, which must not be considered as an every-day pamphlet, may, notwithstanding a few theoretical peculiarities, be considered highly creditable to the talents and industry of its author.

An appendix is attached to the body of the work, consisting of some judicious remarks on rheumatism; but, as we have already exceeded our limits, and, as the author promises to enlarge these hints into the form of a distinct treatise, we shall reserve our remarks till that design is accomplished.—A copious Index is subjoined to the whole, which much enhances the value of a book comprehending the opinions of so many writers who have preceded the author.

---

*Oracular Communications, addressed to Students of the Medical Profession.* By ÆSCULAPIUS. 12mo. pp. 132. Cox and Son, 1816.

THIS whimsical little performance is not destitute of merit and utility; but the whole might have been conveyed in a much less expensive form, and as well as much more compressed. The object is to direct a student in his medical education; and we heartily wish the emoluments of the profession would admit the expences here proposed. We cannot, however, see the necessity of all of them; par-

 ticularly ticularly the journey to Scotland, we conceive, may be dispensed with, as the town which contains the greatest number of inhabitants and the greatest number of hospitals, must exhibit the greatest number of cases, which, in a practical art, is what must be principally wanted. There may be, perhaps, less dissipation, and more habits of application, at Edinburgh; but these may be imitated in London. In London, too, he will be less likely to fall into an error too common among the graduates in the north, and against which the Oracle particularly guards him.

"Above all things, (says he,) let the student avoid becoming the slave of one system. In whatever school of medicine he may be educated, *he will find that only one range of doctrine is accounted orthodox*: let him not adopt these only, as the basis of his *medical creed:* let him recollect, that other schools and other systems are alike built upon classical learning, indefatigable research, and just and solid reasoning,—and let him seek an acquaintance with these systems. This will enlarge his mind, and prevent his sinking at once into the common routine practitioner,—a consequence too often the inevitable result of his reception of one system, his belief of its dogmas, and the discredit he attaches to any valuable information, which may be found any where within the compass of medical science, except within the boundaries prescribed by his own bigotted and sectarian principles. This is a very common mistake; and, when the memory has been stored with these doctrines, the student fancies his knowledge is complete, and slumbers in inactivity, instead of pursuing after fresh and increasing information. Let him recollect that few men think perfectly alike on any subject, and let him seek to obtain an acquaintance with the opinions and practice even of *those* who are excluded by the localities of nature from the little côterie, around which his contracted mind has described the *circle of excellence*, that he may enlarge his views, and, with the information he has acquired, be able to take a comprehensive survey of disease, and to judge of truth for himself. And, to this end, let him gain all possible knowledge of continental medicine. All knowledge will be of use to him: and he should not despise any acquisition which will render him at all more fitted for the practice of his profession."

In another place we meet with some judicious remarks on "continental medicine," as it is called. The author agrees with us, that, though their pathology is extremely deficient, yet, in description of disease, they are frequently more accurate than ourselves.

For those, however, who cannot afford to visit more than one seat of learning, we think it will not be disputed that in London there is less danger from the inevitable result of the reception of only one system, especially if the student follows the advice of one of our correspondents in attending two lecturers in medicine.

*Medico-*

*Medico-Chirurgical Remarks and Observations of the late Dr. M. G. Thilenius, Physician, &c.* Published after his Decease, by his Son, Dr. H. C. Thilenius. Francforth, 1814.

[From our Correspondent in Germany.]

Thilenius, the father, promised, in the second edition of his Medico-Chirurgical Remarks, 1809, to publish a second volume of it, but death prevented the execution; this induced the son to select, arrange, and publish, the volume now before us, from among the papers of the deceased. In doing so, we are told in the preface, he met with many difficulties, for, there being no plan to be found according to which the deceased would have wished it to be executed, it was uncertain what he would have omitted, and what added. There were also by far less materials, as might have been expected, for the author sometimes not having been thoroughly satisfied with what he had written, had often erased it. To fill up these vacancies, and at the same time to complete the work for those that possessed the first volume of the second edition, the editor adhered to the alphabetical order of the first, and added such articles as were deficient in the first part of the second edition, in the same order as in the first edition. The alterations made by the deceased are given unaltered as they were found, and remarkable cases are added from the editor's own diary, as documents to the others. Thus he completed, as well as he could, whatever appeared deficient, marking his own remarks with a (*). The work is thus to be considered in a double view; first, as a new and augmented edition of a work sufficiently known and decidedly valued; and, secondly, in regard of the marked additions, as an entirely new work of the editor himself. To satisfy both these points, each by a separate criticism, would require too long a notice, and be too prolix for the scope of these pages. We must, therefore, confine ourselves to a short and general judgment, assuring our readers, that the additions and alterations of the deceased bear ample testimony of his constantly aiming at superior technical perfection, and that they may be considered as real improvements, as well as that the retouching of, and additions to, most articles by the editor, prove him to be a meritorious son of a celebrated father.

The biography added by a grateful admirer of the deceased, the Rev. Mr. Testor, as well as the list of the publications of the late Dr. T. we shall pass over, and select only a few of the most remarkable and circumstantial articles, adding to these our own observations, enclosed in [ ].

*Cataracta.*—Here the editor gives us a very interesting case, case, in lieu of the schoolmaster at Eicha's insignificant operation, which was in the first edition. While he, with the best success, applied galvanism against a paralytic affection of the facial muscles, of a fortnight's standing, he cured, at the same time, through that operation, a considerable debility of the eyes. [It is certain, that this great remedy has been too quickly and yet immaturely resorted to, as also it has been too soon rejected, without being sufficiently tried.]

*Chorea St. Viti.*—An article entirely altered, increased with documents of well marked histories, and distinguished almost throughout with (*). The difference between involuntary muscular motion and chorea is pointedly described. [We have found, however, many things different from Messrs. T.—we have observed the disorder as often in boys as in girls, have found crudities and worms coexistent with, but not as cause of the disorder. In short, we cannot help saying, that too often the fault is laid to worms, whilst other causes of great moment, as that of the unfolding of puberty, are entirely looked over. The same as dentition is frequently accompanied with epilepsy and convulsions, not on account of the pain caused by the cutting of the teeth, but by congestions towards the head,—so do chorea; involuntary motions, convulsions, and epilepsy, arise in all the various periods of growth, and their treatment then does require the utmost caution. And might it not, in the instances mentioned here, where neither worms nor any other occasional cause could be discovered, but where the disorder arose spontaneously, as a *morbus sine materia.* In particular, in the last mentioned case, where the patient, after suffering involuntary motions, and having been left to himself for many years, got the better of it, during the cure of a broken arm, might not the case belong to this category? The author's treatment is evacuant and anthelmintic in the beginning, and, afterwards, antispasmodic and corroborant. Once the disease had been produced by suppressed perspiration of the feet, and once by retrograded itch.]

*Cœliacous fluxus.*—The author confesses, that, having by this time practised nineteen years longer, during which period he had treated nine more patients of this sort than in the first edition, he has not cured one of them thoroughly. The violent pain in the bowels and the tenesmus are, he thinks, proofs of a slow inflammation, the consideration of which ought to precede the use of astringents. That the causes, course, and phenomena, of the disorder may often vary, is fully proved in this edition, by two new cases, (marked *), besides the case already published in the first edition, edition; these remarks furnish a convincing proof of the author's practical talents.

*Deglutitio impedita.*—To the former cases, two new ones are added. One of them was founded on glandular swellings and sympathetic abdominal affections. It was successfully treated with a decoction of spongia marina, Minderer's spirit, aqua laurocerasi, mustard whey, and vesicatoria; the other, most likely the consequence of the pressure of a rib bent inwards, was not cured. Aged people not seldom bring up their food, mixed with a deal of frothy mucus, whilst in the act of deglutition; in this case, pills made of spongia marina, with mucilage of gum arabic, may give relief, but effects no cure.

*Epilepsy.*—All the cases here related, most of which were cured, were symptomatical. The causes were worms, whose expulsion often required a deal of labour and skill, infarction, indigestion, incipient menstruation, lacteal metastasis before the milk had entered the breasts; onanism, once incurable, because both body and mind were affected; ulcers, dried up or repelled by lead medicines, to re-produce these, Dr. T. had the place deeply cauterized with a red-hot iron, which brought on a perfect suppuration, and which being kept up by proper digestives, the epilepsy did not appear again, though vesication had proved useless. Internally, he gave camphor with essentia fuliginis. Suddenly repelled itch;(*) tapeworm, which, according to Dr. T. jun. never causes epilepsy, unless it be seated in the upper part of the intestines, towards the stomach, and so, by particularly irritating the plexus solaris, disturbs the whole nervous system. It is said to prove its existence in that case, besides the ordinary symptoms, by an involuntary spasmodic stretching of the arms, most generally the left, and by a benumbing pricking and cramp in one or more fingers of that side. A remarkable history of this kind is related, where, however, the tapeworm did not exist. Fright.—Among the specifics to be resorted to, where no apparent cause can be found, the author praises the *cuprum ammoniacale*, with which he succeeded in many cases; also, the valerian. Bark, orange leaves, Deppel's oil, and musk, were less certain in their effect.(*) Zinc flowers, in such increased doses, that they produced nausea, were of more service.(*) Camphor, belladonna, stramonium, and hyosciamus, had no effect at all; and lunar caustic, according to Heim's direction, in two desperate cases, had not the least effect. The dissection of a patient, who had, for many years, laboured under this disorder, concludes this article.

*Febris*

*Febris puerperarum.*—A thoroughly specific, always uniform puerperal fever, and always originating from the same cause, does, according to Dr. T.'s opinion [and our own also] and experience, not exist.

"I have read (says he), with equal curiosity and astonishment, the many books, in which they have overwhelmed us for the last thirty years with hypotheses and quite different methods of treatment of this, what they call peculiar, sort of fever. I often compared what I saw at the sick bed, with what I had read, found it contradictory, and threw many a book aside, displeased and perplexed. Often I found the most striking diversities in contemporary patients. I, therefore, always treated my puerperal patients according to their idiosyncrasies, the predominant morbid constitution of the season, and the characteristical symptoms of the fever; by which method, I fared well, and had but seldom to regret the loss of a patient."

"For the rest (says the author) the fever had generally a gastric, bilious, sometimes a nervous, rarely a purely inflammatory, character, though most of them were tinged with it, which always required the greatest attention. In some, the lochial were stopped; in others, they flowed freely. Some lost entirely all milk from the breasts; in others, its draft remained uninterrupted."

This gives us a plain idea of the author's treatment. Young practitioners will derive much instruction from reading the morbid histories here related, as they contain many capital practical maxims and hints.

*Fluor-albus.*—The causes of this disorder are well pointed out. The disorder is represented as a local affection of the vagina, or the uterus, and sometimes of both at once; of which debility, the digestive organs, (very likely more frequent the consequence than the cause,) and, as the consequences thereof, mucosity, mucous infarctus, (which many physicians, though incorrectly, consider as nonsense,) the weak or irritable nerves, more or less, participate. Though the chronic fluor-albus is not curable by purging, yet cleansing of the first passages is considered as necessary to give full scope to the action of the other remedies; as we often remain in the ambiguous state of mending and getting worse, if the concomitant acrimonies are not previously destroyed. Against arthritic and herpetic acrimonies, the author employs the known remedies; but assists their operation by setons and vesicants. Where the discharge has a disagreeable urinous smell, and in scrofulous subjects, muriatic acid was found useful. For tenacious lymph, a decoction of spongia marina tosta, lime-water, and, when too thin, or combined with great laxity, bark, æthiops antimonialis, and cortex sassafras, were employed. A strong infusion of dulcamara has also, according to Dr. Althof's experience, great effect on all these acrimonies; but, at the same time, attention is to be paid to the stomach, giving corroborants after the first passages are cleansed. The specific effect of catechu Dr. T. does not confirm. Proper injections into the vagina are essential to the cure—(their application saves a deal of time, in recent cases, and in old and inveterate ones nothing can be done without them). Clean water being often not sufficiently active, he generally makes use of lime-water. If the discharge is very thick and tenacious, or if the dried stains in the linen leave an earthy meal-like residue behind, the author recommends a solution of the lapis causticus with opium or calx antimonii sulphuratum. In herpetic acrimony (where the last-mentioned remedy is also of service), and in scrofulous acrimony, he recommends Fordyce's medicine, consisting of calomel and gum arabic, each ʒj, with ℥ij of lime-water, or a solution of sublimate. Wherever he suspects an ulcerated state, the Aqua phagedænica, with a decoction of carrots and liquamen myrrhœ, is used. Sometimes the great irritability and pain of the vagina will not bear any of these injections: in this case, he wishes these impediments to be first removed by injections made of a solution of succus liquiritiæ, (would a decoction of the root not answer this purpose better?) decoction of quince-seeds, and flea-bane, with opium, or thirty or forty drops of Aqua lauro cerasi. If the acrimony is removed in this manner, but the discharge still continues from relaxation, then a solution of Gum. kino in lime-water, or a decoction of oak-bark with sugar of lead, or even a solution of kino, with alum, is to be injected. An unusually thin discharge was removed by a solution of septic stone with mucilage of gum tragacanth. Sponges dipped in the above-named medicines may also be used instead of injections, though they are not so convenient, as they require to be changed frequently, and are thus only applicable at nights, or when travelling. Baths are most useful, both generally and topically applied.

*Hæmorrhagia uteri.*—A most instructive article, even for old practitioners. Dr. T. sen. saved the life of many, when on the point of death, by a solution of ten drops of the genuine oil of cinnamon in ʒi of æther, to ten or fifteen drops every quarter or half hour. By perfect atony of the womb from long-continued hæmorrhages, in consequence of the placenta lying before, Dr. T. jun. brought the uterus to immediate contraction, by pouring æther over his hand, and afterwards introducing it, though the hæmorrhage had been so violent that the patient died within a quarter of an hour.(*)

hour.(*) That considerable pieces of the placenta remaining behind, do not always cause an hæmorrhage, (though generally we believe,) the author shows, by a remarkable morbid history, hinting, at the same time, that, if the cohesion between placenta and uterus be sufficiently separated, the process of putrefaction, thus begun, will destroy the still bleeding vessels, and so prevent a new hæmorrhage; but, if the cohesion be still firm, the living vascular connection between placenta and uterus will continue, and an hæmorrhage infallibly be the consequence.(*) Dangerous uterine hæmorrhages, in petechial and putrid puerperal fevers, are almost always combined with colliquative diarrhœas, and require immediate assistance. Only the internal use of sugar of lead, at one-sixth, or, at farthest, one-fourth of a grain, every two hours, and a decoction of bark, with the extract of logwood and gum kino every intermediate hour, combined with injections of decoct. cinchonæ, with scordium, sage, tormentilla, and alum; and fomenting the abdomen with similar ingredients and a solution of sal. ammoniac in equal parts of vinegar and water, have done wonders in some desperate cases. The chapter contains, also, some capital prescriptions for chronical uterine hæmorrhages, and excessive menstruation.

*Hydrops*(*).—For the last four years, the editor has noticed the particular blackness of the blood in dropsical patients; and found it in ascites more of a blackish brown, as if mixed with red-lead: he therefore gave corroborants, in combination with volatile medicines, or ferruginous remedies, combined with diuretics, as soon as he possibly could. This disorder is always difficult to cure. In respect of the evacuation of the accumulated fluid, a great deal depends upon its nature. For thick tenacious lymph, the author praises the use of mercury, the action of which, it is said, may be still increased, by a combination with a decoction of senega. Ptyalism shews, he thinks, that the lymph has become attenuated, and fit for expectoration; and then he gives the squill.

We beg the candour of our readers for the above article, in which, with some useful practical hints, it must be admitted there is much antiquated pathology.

---

*Dissections*. By D. Godfred Heischman, Private Teacher of Anatomy, and Prosector of the Anatomical Theatre at Erlangen.—Erlangen, 1815. pp. 264. Large 8vo. with one Plate.

This work, in which the author gives proofs of learning, talent, and industry, affords a considerable contribution to morbid anatomy. Under twelve distinct divisions, he gives us the description of ninety-six morbid abnormities, arranging them in the following manner:—I. Abnormities of the intestinal canal. II. Abn. of the stomach. III. Abn. of the liver. IV. Abn. of the kidneys. V. Abn. of the male genitalia. VI. Abn. of the female genitalia. VII. Abn. of the heart and lungs. VIII. Abn. of the bulb of the eye. IX. Abn. of the muscular system. X. Abn. of the vascular system. XI. Abn. of the bones. XII. Abn. of the skin.

---

